# Defining Core competencies of the European Society for Sports Traumatology, knee surgery and arthroscopy

**DOI:** 10.1186/s40634-020-00276-0

**Published:** 2020-07-31

**Authors:** Michael Taylor Ross, Martin Lind

**Affiliations:** 1 Primrose Lane Medical Centre, 3-5 Jutland Street, Rosyth, Fife, UK; 2 Department of Orthopaedic Surgery, Århus University Hospital, Århus, Denmark

**Keywords:** Curriculum, Core, Competencies, Orthopaedics, Sports medicine, Survey

## Abstract

**Purpose:**

The European Society for Sports Traumatology, Knee Surgery and Arthroscopy (ESSKA) identified the need to develop a competency-based core curriculum for ESSKA specialists, against which all their educational activities, resources and priorities for development could be mapped. The aim of this study was to take a research-based approach to developing a competency-based core curriculum for ESSKA specialists.

**Methods:**

A Core Curriculum Working Group, with experts representing the ESSKA Board, Sections and Committees, reviewed existing curricula and literature in their own specialist areas and developed a draft list of 285 core competencies for ESSKA specialists. All ESSKA members were asked to comment and rate the importance of these competencies, and the Working Group used these results to refine the curriculum.

**Results:**

Four hundred-forty responses to the online survey contained meaningful data. Almost all were ESSKA members, with broad representation of the countries, ages and backgrounds of the membership. All 285 core competencies were considered at least ‘Important’ for ESSKA specialists so are retained in the final curriculum, and no new competencies were added. 82 (29%) were considered ‘Essential’, constituting between 19% and 37% of the competencies within each specialist area. 96 (33.5%) were considered ‘Very Important’, and 107 (37.5%) ‘Important’.

**Conclusions:**

A competency-based core curriculum for ESSKA specialists was achieved through a systematic and scholarly approach, involving both expert opinion and engagement of the wider ESSKA membership. The core curriculum addresses the identified need in terms of educational development for ESSKA and should also be of interest to the wider orthopaedic and sports medicine communities.

## Background

The European Society for Sports Traumatology, Knee Surgery and Arthroscopy (ESSKA, www.esska.org) is an international membership organisation for specialists in degenerative joint disease and sports medicine. It brings orthopaedic surgeons and other musculoskeletal specialists together to share best practice, collaborate in research and education, publish journals and other resources, and organise courses and meetings – all with the ultimate goal of improving patient care. For many years, ESSKA’s Education Committee has been reshaping the educational activities provided by the society to its members, including updating and adding surgical skills courses in various specialist areas, developing a prioritised ‘Educational Roadmap’ for ongoing development, and launching the online ‘ESSKA Academy’ platform [1].As part of this process the development of a core curriculum was highlighted as an essential strategic priority, against which all existing educational activities and resources could be mapped, needs-analyses could be undertaken, and gaps and areas for development could be identified. This would then structure and inform all of ESSKAs future educational activities, and could be used as a template for selecting and developing processes for assessment and accreditation, and would also hopefully be of interest and use to those out-with ESSKA.

Core curricula have been defined for many healthcare disciplines, specialities and levels of training. For example, core Learning Outcomes / Competencies have been agreed across Europe for the Bachelor and Master (primary medical degree) in Medicine (e.g. Cumming and Ross 2008; Ross et al. 2014a), for postgraduate training and continuing professional development [2] [3] [4], and for research in medicine and related disciplines up to Doctoral level [5]. Core Learning Outcomes, Competencies, Objectives (often broken-down into Knowledge, Skills and Attitudes), Aims and, increasingly, ‘Entrustable Professional Activities’ (EPAs), are typicially used to define the core content which successful participants should learn during an educational course or programme [6, 7]. Core Competencies, which define what every member of a particular group can (or should) be able to do, can be considered as equivalent to Core Learning Outcomes in relation to a specific educational course or programme at the point of graduation [8] . Competency frameworks are more flexible than the other descriptors, however, as they can also be used to define what members of a group can do who are not associated with a single course or programme, and may have very different backgrounds, training and experience. For example, national Core Competencies have been defined in relation to teaching for all doctors involved in teaching or training [9] Such competency frameworks can be used to ‘map’ (cross-reference) educational events, resources and assessments of individual ability [10]. The International Society on Thrombosis and Haemostasis (ISTH) was one of the first medical societies to gain consensus on core competencies for clinical specialists in thrombosis and hemostasis worldwide [11], and they have now also developed core competencies for laboratory specialists in thrombosis and hemostasis, who have even more diverse backgrounds [12]. Because of the success of the ISTH projects, and similarities between these international societies, the ESSKA Board considered the ISTH approach as a helpful model to inform its own research. This aim of the present study was to develop a competency-based core curriculum for ESSKA specialists, covering all areas of specialist interest within the Society, against which all educational activities, resources and priorities for development could be mapped.

## Methods

Many different approaches have been used to define core curricula - ranging from the opinions of one individual or group of experts, literature review and synthesis, opinion surveys, job analysis, and various other qualitative and quantitative research methods (Harden 1986). For the current study, it was agreed that a sequential approach combining expert group opinion, review and synthesis of existing literature and curricula, followed by a stakeholder survey to consider and rate a draft competency framework, would be most appropriate. Because of the diversity of backgrounds and areas of specialist activity within the ESSKA community, a single framework of core competencies with a modular design was chosen, so that individual members could easily identify the core competencies relevant to their own areas of practice and interest. It was decided that each competency would be defined in terms of the clinical condition or situation and the relevant procedure to manage this, and that further exploration of how and when individuals should achieve these would be deferred for future research. Groups of experts would be selected to research, develop and refine each of the specialist areas of the core curriculum, recognising that it was unlikely that any one individual would have sufficient expertise in all of the specialist areas covered by ESSKA. Managerial approval for the project was secured from the ESSKA Board, who confirmed that no additional ethical approval was required.

### Expert group selection and drafting of curricular modules

The Core Curriculum Working Group was initially constituted of seven expert groups, reflecting the seven main areas of activity within ESSKA, led by the ESSKA Education Secretary (ML) with assistance from a medical educationalist (MR). Each expert group had a nominated member of the ESSKA Board, plus two nominated specialists from each of the following ESSKA Sections and Committees: Knee Arthroscopy (via the Arthroscopy Committee); Degenerative Knee (via the European Knee Association); Hip (via the Hip Committee); Sports Medicine (via the European Sports Medicine Association); Shoulder (via the European Shoulder Association); Foot and Ankle (via the Ankle & Foot Associates Section); and Elbow and Wrist (via the Elbow & Wrist Committee). Many of the expert groups recruited additional members based on their specialist skills and experience. Each expert group worked semi-independently in liaison with the educationalist to review the literature and relevant existing national and international curricula, including the content of existing and forthcoming ESSKA courses and training materials. They also liaised with colleagues and their associated Sections and Committees, and iteratively develop and agree an initial draft of core competencies for their own specialist area. Some documents were found to be relevant to multiple specialist areas and so were shared between them, such as the 2015 EFORT and 2000 AOSSM curricula [13–15], and various national curricula for orthopaedic trainees in Europe and elsewhere. In reviewing and drawing from such curricula, the expert groups were mindful to focus on developing a set of core competencies at an appropriate level for ESSKA members who have completed their specialist training and not, for example, another comprehensive curriculum for orthopaedic or sports medicine trainees.

### Reviewing and synthesizing curricula from each specialist area

After sharing earlier drafts and multiple online meetings, the Working Group met in person to review, discuss and refine the early draft curricula from each of the seven expert groups. Areas of overlap were discussed, there was some movement of competencies between groups, and consensus was reached on formatting issues, nomenclature, scope, level of detail, a common structure and sequence for the curriculum, along with the next steps for refining the drafts and creating a single online stakeholder survey in English. The Working Group agreed to the Elbow & Wrist group’s proposal to focus more specifically on ‘Elbow & Forearm’; that the arthroscopic and degenerative knee groups would combine their efforts and draft curricula and become a single ‘Knee’ group; and that preventive and non-surgical procedures for all anatomical areas would be incorporated by the Sports Medicine group into a single set of ‘Sports & Exercise’ competencies. The resulting six expert groups then continued to work on their own areas of the core curriculum, incorporating the feedback and principles agreed by the Working Group, in liaison with their associated Sections and Committees, until satisfied that their draft list of competencies was ready for wider dissemination and feedback.

### Online survey creation and pilot

A draft online survey was created in SurveyMonkey (www.surveymonkey.com), with the agreed competencies from each of the six expert groups, in the agreed sequence of Sports & Exercise followed by each anatomical area from Shoulder down. Consistent with the aims of the current research, the Working Group prioritised the perceived importance of each competency for ESSKA members, rather than exploring when or to what extent these should be achieved using a variation of Miller’s triangle (Miller 1990). Respondents were therefore initially asked to rate the importance of each competency (defined by the type of condition as a stem question followed by a series of specific procedures) on a 3-point Likert scale, whether they felt there was anything missing or unclear, and some demographic information. Respondents were also asked at what level of training course they would expect to see the competency covered, to guide future educational activities, but these did not inform development of the core competencies and so these questions and data are not reported here. The survey was then reviewed and piloted twice by members of the Working Group and others, who felt that the 3-point Likert scale was not sufficiently discriminatory, that the whole survey was lengthy, and some specialist areas were less relevant to certain individuals. It was therefore decided that stakeholders would be asked to rate the importance of each competency for ESSKA specialists on a 5-point Likert scale (1 = Not Important; 2 = Limited Importance; 3 = Important; 4 = Very Important; 5 = Essential) - both in their own main specialist area and as many of the other areas as they felt able to rate. Additionally, some duplication and overlap between specialist areas was removed – for example, steroid injection for frozen shoulder was removed from Shoulder as it was already covered by ‘Injection therapy’ in Sports & Exercise. There was also some resulting refinement and standardisation of terminology, such as changing ‘upper’ to the more anatomically-correct term, ‘proximal’. The Working Group then agreed a final list of 285 competencies (detailed in Table 2) for inclusion in the online stakeholder survey, with 42 in Sports & Exercise; 67 in Shoulder; 34 in Elbow & Forearm; 41 in Hip; 56 in Knee (combined arthroscopic and degenerative); and 45 in Foot & Ankle.

### Stakeholder survey recruitment and information

**Fig. 1:**
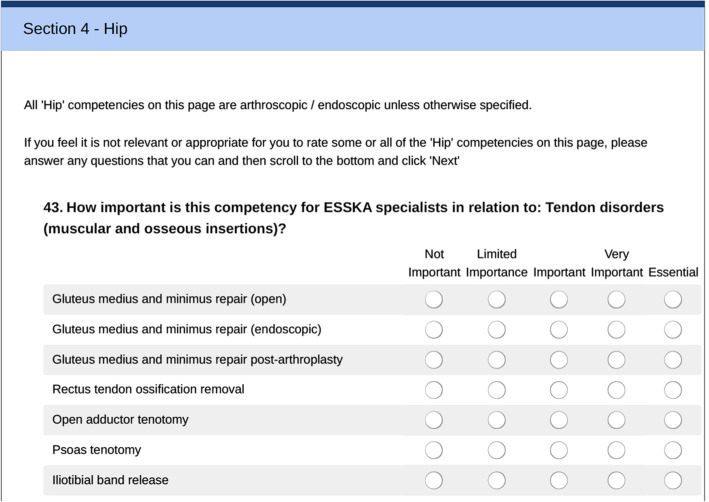
Example screenshot from online survey

An initial e-mail invitation to complete the online survey was sent to all 2954 ESSKA members, as well as 5814 ‘friends of ESSKA’ (former ESSKA members and participants in ESSKA congress, courses or fellowships). Two further reminders were sent to the whole membership, and some people were also directly encouraged to respond by colleagues in the Board or Working Group. Participants were informed about the research including how the draft competencies had been developed, the scope of the curriculum and clarification that it did not include all aspects of an orthopaedic training curriculum nor new and experimental procedures, and ethical issues such as consent and respondent anonymity. It was also made clear that all competencies implicitly assume the specialist has adequate facilities, resources and staff support to undertake the procedure and manage common complications (such as minor post-op infection and bleeding), as well as sufficient prerequisite training both generally (e.g. communication and infection control) and specifically for that procedure (including being able to appropriately assess the patient, select the most appropriate procedure, and have the knowledge and expertise to perform the full procedure safely and successfully). participants were then asked to respond to all questions related to their own specialist area and as many of the other areas as they felt able to rate, as well as some demographic questions. Figure 1 shows a typical screenshot from the online survey.

### Analysis of findings and expert group decisions

After sufficient responses had been collected, the survey was closed and the data were exported, sorted and provisionally analysed in Excel. Responses which did not contain meaningful data were removed, and the ratings were analysed in various ways, including calculating the Mean rating of all responses for each competency, the Mean rating for the subgroup of respondents who specialised in that particular area, and the percentage of all respondents who indicated each competency should be Essential or Very Important. Demographic data were also summarised, and all free-text responses were collated in a single document for analysis. A teleconference was then arranged for each of the six expert groups with the Educationalist to review and make some collective decisions related to the survey findings in each of their specialist areas. First, with the competencies ranked by Mean of all responses, the expert groups were asked to consider how many Likert scale ‘Levels’ these represented, and whether any of the lowest-rated Mean competencies should be removed from the draft list. Second, the expert groups were asked to select an appropriate cut-off between each Level, with the competencies ranked by the percentage of respondents indicating it should be higher than this (i.e. the percentage who rated competencies as 5 for the cut-off below ‘Essential’, and as either 4 or 5 for the cut-off below ‘Very Important’). Third, each expert group also reviewed all the free-text comments to determine whether any competencies should be added or reworded.

## Results

### Respondent demographics

The survey was open from 15th May to 15th August 2019. There were 625 responses in total, 440 of which contained meaningful data. The largest proportion of respondents (43%) indicated that Knee-Arthroscopy best represented their speciality, with smaller numbers indicating Knee–Degenerative (17.4%), Shoulder (14.1%), Sports Medicine (13.8%), Foot & Ankle (5.8%), Hip (2.7%), Elbow & Forearm (0.8%), and ‘Other’ (2.4%, clarified as combinations of the above or related sciences). The average age of respondents was 46, ranging from 26 to 73 years. Respondents were mostly experienced surgeons, with 34.5% indicating they had worked for 11–20 years after completion of training; 23.8% for ≥21 years; 17.4% for 6–10 years; 16.7% for up to 5 years; and 3.6% were still in postgraduate training. There were also small numbers of scientists (2%), physiotherapists (1%) and ‘Others’ (0.7% - clarified as academics). Respondents represented a broad range of ESSKA member nationalities, specifically: Spain and Greece (22% each); Germany (20%); Italy (19%); Poland (14%); Portugal and Romania (both 12%); the UK and Switzerland (9% each); France, Denmark and Turkey (8% each); Belgium and Austria (7% each); Norway and Bulgaria (6% each); the Netherlands, Ukraine, Russia and Japan (5% each); Sweden, Finland and Croatia (5% each). 97% of respondents indicated they were current ESSKA members, with the others being ‘friends of ESSKA’.

### Rating of survey competencies and expert group decisions

As expected from the highly-specialised and modular nature of the draft core curriculum, many respondents left some subject areas of the questionnaire blank. All of the Sports & Exercise competencies, which were presented first in the survey, were rated by at least 400 respondents. The Knee competencies were each rated by at least 238 respondents, and the Shoulder competencies by at least 226 respondents. The other three subject areas had a combined average of 122 ratings per competency, with the lowest being 109 ratings for one of the Foot & Ankle competencies. The lowest Mean rating for any competency by all respondents was 2.73. When the competencies were reviewed by Mean rating in each specialist area, each of the expert groups agreed that the Means could be grouped into three Levels: 3 (Important), 4 (Very Important) and 5 (Essential). They also agreed that all of the draft competencies were considered to be important enough to remain in the final framework. Each expert group identified and agreed appropriate cut-offs in the ranked competencies to define these three Levels, considering both the ratings and the face-validity of the specific competencies on either side of the cut (Table 1).

**Table 1:** Cut-offs between ranked competencies agreed by expert groups

	Cut-off when ranked by:		Resulting competencies at each Level		
	5/n	4 + 5/n	Essential	V. Important	Important
Sports & Exercise	Essen. ≥ 31.0%	Imp. ≤ 39.8%	8 (19%)	17 (40.5%)	17 (40.5%)
Shoulder	Essen. ≥ 33.1%	Imp. ≤ 49.8%	25 (37%)	26 (39%)	16 (24%)
Elbow & Forearm	Essen. ≥ 22.3%	Imp. ≤ 42.2%	11 (32%)	15 (44%)	8 (24%)
Hip	Essen. ≥ 22.0%	Imp. ≤ 45.7%	13 (31.5%)	4 (10%)	24 (58.5%)
Knee	Essen. ≥ 47.1%	Imp. ≤ 58.2%	15 (27%)	21 (37.5%)	20 (35.5%)
Foot & Ankle	Essen. ≥ 35.8%	Imp. ≤ 56.8%	10 (22%)	13 (29%)	22 (49%)

Table 2 shows the full list of 285 competencies, arranged by Mean rating for all responses within each specialist area. It also shows the Mean rating for the subgroup of respondents who indicated that this was in their own specialist area for comparison; the percentage of all respondents who rated each competency as Essential (5/n); the percentage of all respondents who rated them as either Very Important or Essential (4 + 5/n); and the Level these were allocated-to by each expert group based on the cut-offs they had agreed. Of note, there were only three competencies where the Mean rating of area specialists was more than one point of difference from the Mean of all responses, which have been highlighted in bold in Table 2.

**Table 2:** For insertion in the results section as indicated above

	Mean all responses	Mean for specialists	Essential % (5/n)	E + VI % (5 + 4/n)	Level
**Sports & Exercise**					
Acute muscle and tendon injury: Direct tendon repair	4.14	4.15	40.9%	77.9%	Essential
Acute muscle and tendon injury: Tendon Anchor reinsertion	4.07	3.96	36.4%	75.7%	Essential
Emergencies on the field: Fracture / dislocation reduction / realignment, immobilization and analgesia	3.99	4.14	37.5%	71.6%	Essential
Emergencies on the field: Fracture / dislocation identification, removal from play & assessment	3.95	4.22	36.8%	68.4%	Essential
Return to sports: Muscle strength evaluation	3.86	4.02	26.3%	67.1%	Very Important
Acute muscle and tendon injury: Tendon reinforcement plasty	3.79	3.79	25.7%	63.8%	Very Important
Emergencies on the field: Concussion identification, removal from play & assessment	3.74	4.04	31.0%	60.3%	Essential
Rehabilitation: Injection therapy (corticosteroids; hyaluronic acid; platelet-rich plasma; collagen)	3.73	3.94	25.1%	57.3%	Very Important
Acute muscle and tendon injury: Fasciotomy for acute compartment syndrome	3.72	3.88	32.0%	57.8%	Essential
Emergencies on the field: Spinal injury identification, assessment, spinal immobilization and extrication	3.68	4.12	35.1%	57.7%	Essential
Rehabilitation: Exercise therapy	3.65	3.84	23.8%	56.3%	Very Important
Emergencies on the field: Basic life support and automated external defibrillation (AED)	3.62	4.06	35.2%	55.7%	Essential
Chronic muscle and tendon injury (including overuse): Debridement and tendon revision / augmentation	3.61	3.73	20.1%	54.7%	Very Important
Rehabilitation: Functional rehabilitation	3.60	3.70	22.9%	53.9%	Very Important
Return to sports: On the field exercises and tests	3.60	3.92	22.0%	56.3%	Very Important
Chronic muscle and tendon injury (including overuse): Tendon transfer	3.49	3.60	18.8%	51.8%	Very Important
Promoting health and preventing problems: Preventing, identifying and addressing doping in sports	3.41	3.62	25.3%	46.7%	Very Important
Emergencies on the field: Advanced Life Support	3.40	3.80	23.9%	48.9%	Very Important
Return to sports: Isokinetic evaluation	3.39	3.69	14.0%	46.5%	Very Important
Chronic muscle and tendon injury (including overuse): Tendon lengthening	3.38	3.40	16.3%	44.6%	Very Important
Rehabilitation: Use of orthoses	3.37	3.53	13.7%	42.3%	Very Important
Promoting health and preventing problems: Preventing cardiac and other sudden death in sports	3.31	3.64	23.7%	43.3%	Very Important
Promoting health and preventing problems: Advising athletes on preventing and managing fatigue	3.30	3.60	13.5%	41.2%	Very Important
Bursitis, tendinopathy and muscle fibrosis: Ultrasound-guided muscle / bursa / tendon injection	3.26	3.74	11.1%	40.7%	Very Important
Chronic muscle and tendon injury (including overuse): Tenotomies	3.25	3.54	14.3%	39.8%	Important
Chronic muscle and tendon injury (including overuse): Fasciotomy for chronic compartment syndrome	3.22	3.60	12.9%	39.0%	Important
Intra-articular conditions (e.g. hemarthrosis, synovitis): Ultrasound-guided intra-articular aspiration-injection	3.20	3.46	16.4%	43.5%	Very Important
Promoting health and preventing problems: Advising athletes on chronic disease management	3.17	3.46	8.6%	36.8%	Important
Acute muscle and tendon injury: Muscle haematoma drainage	3.10	3.33	10.3%	33.4%	Important
Rehabilitation: Manual therapy	3.09	3.27	9.4%	34.5%	Important
Acute muscle and tendon injury: Direct muscle repair	3.07	3.20	10.6%	35.8%	Important
Promoting health and preventing problems: Kinesiophobia prevention, identification and management	3.01	3.34	10.1%	31.9%	Important
Rehabilitation: Rest / passive therapy	2.99	3.20	8.7%	30.3%	Important
Chronic muscle and tendon injury (including overuse): Myositis ossificans excision	2.98	3.14	8.2%	27.1%	Important
Chronic muscle and tendon injury (including overuse): Fibrotic tissue excision	2.92	3.08	6.3%	24.6%	Important
Return to sports: Electromyography evaluation	2.88	3.02	6.5%	24.4%	Important
Promoting health and preventing problems: Advising athletes on nutrition	2.87	3.23	5.9%	27.3%	Important
Promoting health and preventing problems: Kinesiophobia assessment using the Tampa Scale	2.78	3.16	5.3%	21.3%	Important
Rehabilitation: Aquatic therapy	2.77	3.04	5.6%	21.4%	Important
Rehabilitation: Extracorporeal shockwave therapy	2.75	3.00	5.5%	19.7%	Important
Chronic muscle and tendon injury (including overuse): Muscular pseudo-cyst excision	2.74	2.92	5.3%	19.1%	Important
Rehabilitation: Electrotherapy	2.73	3.00	6.7%	20.1%	Important
**Shoulder**					
Glenohumeral instability: Arthroscopic anterior repair (labrum, capsule)	4.38	4.76	57.7%	85.4%	Essential
Rotator cuff tears: Rotator cuff repair of full thickness tear (arthroscopic)	4.37	4.75	56.0%	86.4%	Essential
Rotator cuff tears: Subscapularis repair (arthroscopic)	4.27	4.60	47.5%	83.8%	Essential
Rotator cuff tears: Partial rotator cuff repair (arthroscopic)	4.20	4.49	47.5%	78.9%	Essential
Glenohumeral instability: Arthroscopic posterior repair (labrum, capsule)	4.16	4.49	45.6%	78.1%	Essential
Biceps tendon disorders: Biceps tenodesis (arthroscopic)	4.13	4.40	42.7%	76.5%	Essential
Biceps tendon disorders: Biceps tenotomy (arthroscopic)	4.08	4.40	43.9%	73.4%	Essential
Glenohumeral instability: Open coracoid transfer procedure	4.06	4.48	41.1%	72.5%	Essential
Joint infections (including Cutibacterium Acnes): Arthroscopic joint debridement	4.06	4.43	42.7%	70.9%	Essential
Rotator cuff tears: Takedown and repair of partial tear	4.05	4.36	37.2%	75.3%	Essential
Frozen shoulder: Arthroscopic capsular release	4.05	4.29	40.6%	72.6%	Essential
Joint infections (including Cutibacterium Acnes): Tissue sample collection for cultivation	4.05	4.44	43.4%	68.5%	Essential
Glenohumeral instability: Arthroscopic anterior augmentation procedures	4.03	4.26	40.6%	71.8%	Essential
Biceps tendon disorders: SLAP tear fixation (arthroscopic)	4.02	4.19	37.2%	73.6%	Essential
Acromioclavicular (AC) joint dislocation: Open reconstruction procedures (chronic)	4.01	4.32	33.1%	74.2%	Essential
Rotator cuff tears: Reverse shoulder arthroplasty	4.00	4.46	38.8%	72.6%	Essential
Subacromial impingement syndrome: Acromioplasty (arthroscopic)	3.99	4.19	45.1%	65.1%	Essential
Rotator cuff tears: Trans-tendon repair of partial tear	3.98	4.17	36.6%	70.8%	Essential
Glenohumeral instability: Arthroscopic remplissage	3.97	4.24	34.6%	70.9%	Essential
Acromioclavicular (AC) joint dislocation: Open fixation (acute)	3.97	4.21	37.0%	71.9%	Essential
Subacromial impingement syndrome: Bursectomy (arthroscopic)	3.97	4.11	44.3%	66.8%	Essential
Rotator cuff arthropathy: Reverse shoulder arthroplasty (RSA)	3.97	4.59	42.3%	68.4%	Essential
Osteoarthritis: Glenohumeral arthroplasty (anatomic / reverse)	3.94	4.54	37.8%	67.0%	Essential
Glenohumeral instability: Open bone graft procedure (anterior, posterior)	3.85	4.24	31.5%	64.7%	Very Important
Calcifying tendinitis: Calcium deposit removal (arthroscopic)	3.84	3.90	26.9%	64.7%	Very Important
Acromioclavicular (AC) joint dislocation: Arthroscopic-assisted fixation (acute)	3.83	4.16	33.8%	64.6%	Essential
Rotator cuff tears: Graft augmentation for irreparable cuff lesions	3.82	3.98	30.7%	64.7%	Very Important
Acromioclavicular (AC) joint dislocation: Arthroscopic-assisted reconstruction procedures (chronic)	3.80	4.13	29.1%	63.2%	Very Important
Subacromial impingement syndrome: Coracoacromial ligament release (arthroscopic)	3.79	3.90	35.0%	61.5%	Essential
Articular cartilage and sub-chondral bone disorders: Distal clavicle excision (arthroscopic)	3.77	4.06	28.1%	62.6%	Very Important
Rotator cuff tears: Tendon transfers (open)	3.74	3.97	26.0%	59.6%	Very Important
Hardware breakage or disengagement: Removal of materials (arthroscopic)	3.74	4.02	28.9%	58.2%	Very Important
Rotator cuff tears: Rotator cuff repair of full thickness tear (open)	3.72	3.54	32.5%	60.4%	Very Important
Biceps tendon disorders: Biceps tenodesis (open)	3.72	3.79	28.8%	60.1%	Very Important
Hardware breakage or disengagement: Removal of materials (open)	3.69	4.00	30.3%	54.7%	Very Important
Rotator cuff tears: Head depressing procedures (arthroscopic, e.g. balloon, superior capsule reconstruction, etc.)	3.67	3.81	24.7%	57.4%	Very Important
Subacromial impingement syndrome: Coplaning acromioclavicular joint (arthroscopic)	3.67	3.72	30.1%	57.2%	Very Important
Nerve and neuromuscular disorders: Scapular dyskinesis rehabilitation	3.65	3.89	26.4%	55.8%	Very Important
Glenohumeral instability: Arthroscopic bone graft procedure (anterior, posterior)	3.64	3.81	25.5%	55.3%	Very Important
Osteoarthritis: Glenohumeral hemiarthroplasty	3.63	4.00	25.9%	54.3%	Very Important
Rotator cuff tears: Tendon transfers (arthroscopic)	3.62	3.75	24.2%	56.4%	Very Important
Articular cartilage and sub-chondral bone disorders: Arthroscopic debridement for chondrolysis	3.62	3.73	24.5%	53.2%	Very Important
Glenohumeral instability: Open anterior repair (labrum, capsule)	3.61	3.54	27.8%	57.0%	Very Important
Nerve and neuromuscular disorders: Winging scapula rehabilitation	3.60	3.85	23.9%	53.9%	Very Important
Fractures: Arthroscopic reduction and fixation of intra-articular glenoid fractures	3.59	3.76	23.3%	51.7%	Very Important
Fractures: Arthroscopic reduction and fixation of tuberosity fractures	3.54	3.67	24.2%	49.8%	Important
Rotator cuff tears: Head depressing procedures (open, e.g. balloon, superior capsule reconstruction, etc.)	3.52	3.40	22.8%	51.5%	Very Important
Frozen shoulder: Mobilisation under anaesthesia	3.52	3.52	26.2%	52.8%	Very Important
Osteoarthritis: Arthroscopic glenohumeral capsular release and joint debridement	3.52	3.84	24.0%	49.8%	Important
Glenohumeral instability: Arthroscopic McLaughlin procedure	3.50	3.56	19.9%	48.2%	Important
Glenohumeral instability: Open McLaughlin procedure	3.50	3.66	19.9%	50.0%	Very Important
Rotator cuff tears: Partial rotator cuff repair (open)	3.48	3.24	25.2%	52.5%	Very Important
Glenohumeral instability: Arthroscopic coracoid transfer procedure	3.48	3.46	24.1%	51.1%	Very Important
Glenohumeral instability: Open posterior repair (labrum, capsule)	3.45	3.30	23.6%	50.2%	Very Important
Nerve and neuromuscular disorders: Nerve release around the shoulder	3.44	3.71	20.3%	42.4%	Important
Articular cartilage and sub-chondral bone disorders: Arthroscopic-assisted core decompression for avascular necrosis of humeral head	3.39	3.52	17.4%	44.9%	Important
Rotator cuff arthropathy: Cuff tear arthropathy (CTA) prosthesis	3.39	3.46	20.2%	44.7%	Important
Rotator cuff arthropathy: Large hemiarthroplasty	3.27	3.40	15.5%	41.6%	Important
Articular cartilage and sub-chondral bone disorders: Distal clavicle excision (open)	3.26	3.25	16.2%	43.6%	Important
Sterno-clavicular instability / dislocation: Open reduction and fixation (acute)	3.23	3.44	15.9%	37.8%	Important
Sterno-clavicular instability / dislocation: Open reconstruction procedures (chronic)	3.17	3.42	13.0%	34.6%	Important
Osteoarthritis: Glenohumeral fusion (arthrodesis)	2.94	2.94	12.9%	31.9%	Important
Subacromial impingement syndrome: Acromioplasty (open)	2.93	2.65	15.1%	34.9%	Important
Subacromial impingement syndrome: Coplaning acromioclavicular joint (open)	2.91	2.57	14.0%	32.3%	Important
Subacromial impingement syndrome: Bursectomy (open)	2.88	2.70	15.5%	34.1%	Important
Subacromial impingement syndrome: Coracoacromial ligament release (open)	2.86	2.62	13.8%	31.9%	Important
Frozen shoulder: Open capsular release	2.78	2.48	12.6%	29.9%	Important
**Elbow & Forearm**					
Osteochondritis dissecans: Arthroscopic debridement +/− microfracturing and loose body removal	3.88	3.00	29.5%	67.4%	Essential
Simple posterolateral rotatory (PLRI) or medial elbow instability (acute, subacute & chronic): Open ligament repair and / or reconstruction	3.86	4.00	33.3%	62.9%	Essential
Complex Posterolateral / Posteromedial instability of elbow (acute & chronic): ORIF bone injuries +/− graft + ligament repair +/− grafting	**3.80**	**5.00**	29.0%	63.4%	Essential
Ulnar nerve / radial tunnel / pronator syndrome: Release	3.79	4.00	26.7%	60.3%	Essential
Stiff elbow (excluding osteoarthritis): Arthroscopic arthrolysis	3.73	4.00	26.9%	59.2%	Essential
Ulnar nerve / radial tunnel / pronator syndrome: Transposition	3.71	4.00	23.8%	59.2%	Essential
Osteochondritis dissecans: Arthroscopic fixation	3.65	3.00	22.3%	56.2%	Essential
Complex Posterolateral / Posteromedial instability of elbow (acute & chronic): Radial head arthroplasty	3.64	4.00	23.1%	57.7%	Essential
Radiohumeral osteoarthritis: Arthroscopic synovectomy, debridement, arthrolysis, removal of osteophytes or loose bodies	3.63	4.00	20.8%	53.8%	Very Important
Ulnohumeral osteoarthritis: Arthroscopic synovectomy, debridement, arthrolysis, removal of osteophytes or loose bodies	3.60	4.00	22.1%	53.4%	Very Important
Simple posterolateral rotatory (PLRI) or medial elbow instability (acute, subacute & chronic): Arthroscopic ligament repair and / or reconstruction	3.59	4.00	23.1%	52.3%	Essential
Plica syndrome / synovial fringe: Arthroscopic resection	3.58	3.00	26.2%	53.8%	Essential
Osteochondritis dissecans: Open osteochondral graft / mosaicplasty	3.56	3.00	18.8%	52.3%	Very Important
Osteochondritis dissecans: Arthroscopy mosaicplasty / MACI	3.56	3.00	20.8%	52.3%	Very Important
Stiff elbow (excluding osteoarthritis): Open arthrolysis	3.56	4.00	23.6%	48.8%	Essential
Simple posterolateral rotatory (PLRI) or medial elbow instability (acute, subacute & chronic): External fixation	3.52	4.00	21.8%	51.1%	Very Important
Complex Posterolateral / Posteromedial instability of elbow (acute & chronic): External fixation	3.50	4.00	20.2%	52.7%	Very Important
Osteochondritis dissecans: Open fixation	3.50	3.00	20.3%	48.4%	Very Important
Radiohumeral osteoarthritis: Radial head resection +/− soft tissue interposition	3.48	4.00	17.6%	48.1%	Very Important
Radiohumeral osteoarthritis: Radial head / radiocapitellar arthroplasty	3.47	3.00	16.0%	50.4%	Very Important
Snapping triceps: Triceps release / resection and ulnar nerve transposition	3.45	3.00	19.5%	42.2%	Important
Simple posterolateral rotatory (PLRI) or medial elbow instability (acute, subacute & chronic): Arthroscopic lateral plication	3.43	4.00	19.5%	49.2%	Very Important
Radioulnar joint instability (distal or proximal): Soft tissue reconstruction +/− osteotomy	3.41	3.00	13.4%	44.9%	Very Important
Ulnohumeral osteoarthritis: Open synovectomy, debridement, arthrolysis, removal of osteophytes or loose bodies	3.38	4.00	18.0%	43.8%	Very Important
Arthroplasty complications: Single or two stage revision	3.36	4.00	18.1%	44.9%	Very Important
Arthroplasty complications: Triceps reconstruction +/− allograft	3.35	4.00	16.5%	42.5%	Very Important
Complex Posterolateral / Posteromedial instability of elbow (acute & chronic): Total elbow arthroplasty	3.33	3.00	19.5%	41.4%	Important
Radiohumeral osteoarthritis: Open synovectomy, debridement, arthrolysis, removal of osteophytes or loose bodies	3.33	3.00	16.2%	42.3%	Very Important
Ulnohumeral osteoarthritis: Total elbow arthroplasty	3.32	4.00	17.1%	41.9%	Important
Chronic Essex-Lopresti injury: Shortening osteotomy ulna	3.29	3.00	15.9%	39.7%	Important
Arthroplasty complications: Linking of prosthesis	3.23	4.00	13.4%	38.6%	Important
Arthroplasty complications: Removal of prosthesis +/− allograft tendon interposition	3.23	3.00	15.6%	39.1%	Important
Chronic Essex-Lopresti injury: Reconstruction of the interosseous ligament / membrane with allograft or autograft	3.12	4.00	12.0%	35.2%	Important
Ulnohumeral osteoarthritis: Arthrodesis	**3.02**	**2.00**	11.7%	32.8%	Important
**Hip**					
Capsulo-ligamentous disorders: Labral repair-reattachment	3.90	4.60	30.0%	64.6%	Essential
Synovial disorders: Loose body removal	3.86	4.20	28.9%	63.3%	Essential
Synovial disorders: Joint lavage and debridement	3.74	4.00	29.1%	55.9%	Essential
Synovial disorders: Synovial biopsy	3.73	4.30	29.4%	55.6%	Essential
Capsulo-ligamentous disorders: Labral reconstruction	3.72	3.60	23.8%	57.1%	Essential
Articular cartilage and sub-chondral bone disorders: Chondral debridement-abrasion	3.72	4.30	26.9%	55.4%	Essential
Synovial disorders: Synovectomy	3.72	4.30	27.8%	53.2%	Essential
Capsulo-ligamentous disorders: Labral debridement	3.71	3.80	22.0%	58.3%	Essential
Tendon disorders (muscular and osseous insertions): Hamstring repair/reinsertion (open)	3.66	3.70	22.6%	52.6%	Essential
Articular cartilage and sub-chondral bone disorders: Microfracture	3.65	3.80	26.2%	52.3%	Essential
Bony deformities: Femoral osteochondroplasty	3.65	4.40	23.6%	53.7%	Essential
Bony deformities: Acetabular rim trimming	3.62	4.50	23.0%	54.9%	Essential
Synovial disorders: Intra-articular biopsy / hip assessment (post arthroplasty)	3.55	4.20	23.4%	48.4%	Essential
Tendon disorders (muscular and osseous insertions): Iliotibial band release	3.54	3.10	18.1%	48.8%	Very Important
Capsulo-ligamentous disorders: Capsular plication and repair	3.48	3.60	16.1%	47.6%	Very Important
Synovial disorders: Trochanteric bursectomy and spur removal	3.48	3.00	19.2%	43.2%	Important
Bony deformities: Subspine impingement decompression	**3.46**	**4.50**	20.3%	44.9%	Important
Articular cartilage and sub-chondral bone disorders: Scaffold enhanced microfracture (AMIC)	3.44	3.40	18.3%	48.1%	Very Important
Bony deformities: Femoral head decompression for avascular necrosis	3.43	3.10	19.3%	45.4%	Important
Tendon disorders (muscular and osseous insertions): Gluteus medius and minimus repair (open)	3.41	3.40	12.9%	47.7%	Very Important
Bony deformities: Wall fracture osteosynthesis	3.40	3.50	18.8%	42.7%	Important
Tendon disorders (muscular and osseous insertions): Open adductor tenotomy	3.39	2.80	16.5%	43.3%	Important
Tendon disorders (muscular and osseous insertions): Psoas tenotomy	3.39	3.56	12.9%	42.7%	Important
Synovial disorders: Removal of cement / Loose Body (post arthroplasty)	3.38	3.90	16.3%	43.1%	Important
Tendon disorders (muscular and osseous insertions): Gluteus medius and minimus repair post-arthroplasty	3.36	3.60	13.4%	45.7%	Important
Tendon disorders (muscular and osseous insertions): Hamstring repair/reinsertion (endoscopic)	3.31	2.80	18.6%	41.1%	Important
Bony deformities: Ischiofemoral impingement decompression	3.31	3.80	16.0%	37.0%	Important
Tendon disorders (muscular and osseous insertions): Gluteus medius and minimus repair (endoscopic)	3.28	3.20	11.0%	41.7%	Important
Articular cartilage and sub-chondral bone disorders: Chondrocyte transplantation	3.26	3.30	18.6%	39.5%	Important
Bony deformities: Os acetabuli removal	3.25	4.10	17.8%	36.4%	Important
Sciatic nerve entrapment: Sciatic nerve release	3.24	3.00	14.5%	35.9%	Important
Articular cartilage and sub-chondral bone disorders: Open femoral head mosaicplasty	3.22	2.80	20.2%	39.5%	Important
Bony deformities: Open periacetabular osteotomy	3.21	3.20	12.8%	37.6%	Important
Bony deformities: Open proximal femoral osteotomy	3.21	3.30	14.4%	33.9%	Important
Bony deformities: Open femoral osteochondroplasty	3.20	3.60	16.0%	35.3%	Important
Tendon disorders (muscular and osseous insertions): Rectus tendon ossification removal	3.17	2.90	9.6%	40.0%	Important
Tendon disorders (muscular and osseous insertions): Gluteus maximus tendon release (Polesello)	3.15	2.50	11.3%	35.5%	Important
Bony deformities: Os acetabuli fixation	3.13	3.70	15.4%	31.6%	Important
Bony deformities: Open acetabular rim trimming	3.12	3.50	14.3%	32.8%	Important
Capsulo-ligamentous disorders: Ligamentum teres reconstruction	3.11	2.30	11.4%	35.8%	Important
Bony deformities: Reverse open periacetabular osteotomy	3.05	3.40	12.6%	31.1%	Important
**Knee**					
Ligament lesion: Anterior cruciate ligament (ACL) reconstruction	4.65	4.65	74.7%	92.5%	Essential
Meniscal tears (all arthroscopic): Meniscal repair	4.64	4.67	71.3%	93.6%	Essential
Patellofemoral instability: Medial patellofemoral ligament (MPFL) reconstruction	4.47	4.54	61.9%	86.6%	Essential
Meniscal tears (all arthroscopic): Meniscal root repair	4.46	4.51	58.4%	89.6%	Essential
Ligament lesion: ACL revision reconstruction	4.42	4.46	57.6%	86.8%	Essential
Ligament lesion: Lateral collateral ligament (LCL) reconstruction	4.35	4.38	52.0%	86.5%	Essential
Ligament lesion: Posterolateral corner (PLC) reconstruction	4.35	4.42	54.0%	83.7%	Essential
Meniscal tears (all arthroscopic): Partial meniscectomy	4.33	4.35	56.6%	82.5%	Essential
Meniscal tears (all arthroscopic): Meniscal RAMP repair	4.32	4.36	51.4%	84.7%	Essential
Ligament lesion: Medial collateral ligament (MCL) reconstruction	4.26	4.33	49.0%	81.0%	Essential
Ligament lesion: Posterior cruciate ligament (PCL) reconstruction	4.25	4.30	46.8%	81.7%	Very Important
Osteochondritis dissecans: Osteochondral fixation	4.23	4.28	45.8%	79.9%	Very Important
Patellofemoral instability: Tibial tubercle osteotomy	4.20	4.24	47.4%	76.9%	Essential
Ligament lesion: Multi-ligament (2 or more) reconstruction	4.19	4.29	45.2%	78.2%	Very Important
Ligament lesion: Medial collateral ligament (MCL) repair	4.18	4.24	47.4%	78.3%	Essential
Ligament lesion: Paediatric ACL reconstruction	4.16	4.18	45.2%	76.6%	Very Important
Chondropathies: Microfracture	4.14	4.09	47.2%	72.0%	Essential
Patellofemoral instability: Paediatric medial patellofemoral ligament (MPFL) reconstruction	4.14	4.21	48.2%	73.5%	Essential
Bone deformities / malalignment: Proximal tibial osteotomy (2-plane correction)	4.11	4.15	43.3%	74.5%	Very Important
Bone deformities / malalignment: Proximal tibial osteotomy (1-plane correction)	4.10	4.12	45.2%	74.2%	Very Important
Tibiofemoral arthritis: Tricompartmental (Simple) arthroplasty	4.05	4.12	47.1%	71.1%	Essential
Tibiofemoral arthritis: Medial unicompartmental arthroplasty	4.04	4.10	41.6%	71.8%	Very Important
Post-arthroplasty complications: First-stage revision (infection)	4.04	4.07	42.1%	71.7%	Very Important
Bone deformities / malalignment: Distal femoral osteotomy (2-plane correction)	4.00	4.07	39.7%	68.8%	Very Important
Bone deformities / malalignment: Distal femoral osteotomy (1-plane correction)	3.98	4.00	40.3%	67.7%	Very Important
Chondropathies: Arthroscopic debridement	3.96	3.87	44.7%	66.8%	Very Important
Post-arthroplasty complications: Second-stage revision	3.96	4.04	39.0%	68.9%	Very Important
Post-arthroplasty complications: Single-stage revision	3.92	3.95	35.8%	66.7%	Very Important
Chondropathies: Autologous osteochondral transfer	3.90	3.91	34.1%	66.7%	Very Important
Tibiofemoral arthritis: Tricompartmental (Complex) arthroplasty	3.88	3.99	36.4%	66.1%	Very Important
Ligament lesion: Lateral extra-articular tenodesis	3.87	3.92	36.4%	66.4%	Very Important
Bone deformities / malalignment: Osteotomy combined with ligament reconstruction or arthroplasty	3.87	3.92	33.2%	66.0%	Very Important
Synovial disorders: Total synovectomy +/− posterior compartments (arthroscopic)	3.86	3.90	36.3%	62.9%	Very Important
Synovial disorders: Partial synovectomy (arthroscopic)	3.85	3.88	36.0%	60.7%	Very Important
Ligament lesion: Anterolateral ligament (ALL) reconstruction	3.77	3.82	34.1%	62.7%	Very Important
Chondropathies: Scaffold chondral repair	3.76	3.73	29.5%	61.0%	Very Important
Chondropathies: Osteochondral allografting	3.74	3.79	30.5%	57.4%	Important
Bone deformities / malalignment: Double osteotomy (1-plane correction)	3.72	3.76	29.3%	58.1%	Important
Patellofemoral instability: Trochleoplasty (open)	3.69	3.75	29.7%	54.9%	Important
Chondropathies: Autologous chondrocyte transplantation (ACT)	3.68	3.69	27.1%	58.2%	Important
Tibiofemoral arthritis: Lateral unicompartmental arthroplasty	3.67	3.71	28.5%	55.0%	Important
Post-arthroplasty complications: Exploratory arthroscopy	3.66	3.65	31.8%	55.6%	Important
Tibiofemoral arthritis: Bicompartmental arthroplasty	3.63	3.59	30.9%	56.4%	Important
Chondropathies: Autologous bone marrow transplantation	3.61	3.62	26.2%	54.4%	Important
Patellofemoral instability: Rotational osteotomy	3.59	3.68	25.9%	52.2%	Important
Post-arthroplasty complications: Vacuum dressing	3.58	3.61	26.7%	50.0%	Important
Meniscal tears (all arthroscopic): Allograft meniscal transplantation	3.55	3.60	28.3%	49.0%	Important
Patellofemoral degeneration / osteoarthritis: Lateral facetectomy (open)	3.55	3.54	25.6%	53.3%	Important
Patellofemoral degeneration / osteoarthritis: Patellofemoral arthroplasty	3.53	3.58	24.0%	50.0%	Important
Patellofemoral degeneration / osteoarthritis: Lateral facetectomy (arthroscopic)	3.42	3.41	23.4%	46.7%	Important
Meniscal tears (all arthroscopic): Synthetic meniscal implant implantation	3.37	3.37	26.6%	44.8%	Important
Patellofemoral degeneration / osteoarthritis: Small implant cartilage replacement	3.36	3.34	21.2%	44.8%	Important
Patellofemoral instability: Trochleoplasty (arthroscopic)	3.31	3.32	23.3%	43.3%	Important
Post-arthroplasty complications: Arthrodesis	3.26	3.31	19.3%	38.7%	Important
Synovial disorders: Synovectomy (open)	3.17	3.19	21.5%	38.2%	Important
Post-arthroplasty complications: Above-knee amputation	3.00	3.07	17.2%	30.3%	Important
**Foot & Ankle**					
Impingement syndromes: Anterior arthroscopic release of impingement	4.21	4.73	44.6%	77.7%	Essential
Osteochondritis dissecans (OCD): Anterior arthroscopic debridement & microfracture	4.20	4.60	46.7%	77.5%	Essential
Osteochondritis dissecans (OCD): Anterior arthroscopic filling & fixing / grafting	4.11	4.20	41.2%	73.9%	Essential
Impingement syndromes: Posterior arthroscopic treatment of impingement	4.10	4.67	38.3%	74.2%	Essential
Osteochondritis dissecans (OCD): Posterior arthroscopic debridement & microfracture	4.07	4.47	42.4%	70.3%	Essential
Lateral ankle ligament injuries: Open repair and reconstruction	4.05	4.47	39.3%	73.8%	Essential
Osteochondritis dissecans (OCD): Posterior arthroscopic filling & fixing / grafting	3.99	4.07	36.4%	66.9%	Essential
Lateral ankle ligament injuries: Arthroscopic repair and (minimally-invasive) reconstruction	3.95	4.40	39.2%	62.5%	Essential
Tendon disorders: Haglund resection	3.94	4.27	36.6%	61.0%	Essential
Tendon disorders: Open surgical procedures (Flap plasties, flexor hallucis longus transfer) for chronic Achilles rupture	3.91	4.07	29.1%	67.5%	Very Important
Tendon disorders: Non-operative management of tendon disorders	3.91	4.25	35.8%	62.5%	Essential
Loose bodies and fractures: Os trigonum removal (posterior arthroscopy)	3.90	4.60	33.6%	63.9%	Very Important
Loose bodies and fractures: Arthroscopic removal of talocrural loose bodies (anterior / posterior ankle)	3.89	4.27	31.6%	64.1%	Very Important
Complications of surgery: Managing tendon injuries	3.89	4.00	28.6%	67.0%	Very Important
Syndesmosis injuries: Arthroscopic syndesmotic repair for acute injury	3.82	4.07	31.4%	63.6%	Very Important
Impingement syndromes: Arthroscopic (posterior + sinus tarsi) release of subtalar impingement	3.81	3.93	25.6%	62.4%	Very Important
Lateral ankle ligament injuries: Use of orthoses	3.78	3.80	29.8%	57.3%	Very Important
Syndesmosis injuries: Arthroscopic reconstruction for chronic injury	3.78	3.73	30.8%	61.7%	Very Important
Tendon disorders: Retrocalcaneal bursectomy	3.77	4.07	26.7%	54.3%	Important
Complications of surgery: First-stage revision (infection)	3.75	3.73	28.3%	60.2%	Very Important
Loose bodies and fractures: Arthroscopic removal of subtalar loose bodies / missed fractures	3.74	4.00	25.4%	58.8%	Very Important
Tendon disorders: Minimal invasive & endoscopic-assisted sutures for Achilles tendon rupture	3.72	4.00	29.9%	53.8%	Important
Degenerative disorders / osteoarthritis: Arthroscopic fusion of subtalar joint (combined posterior & subtalar approach)	3.72	4.13	20.7%	59.5%	Very Important
Complications of surgery: Conservative management of post-arthroscopy pain	3.72	3.80	26.8%	58.0%	Very Important
Complications of surgery: Operative wound revision – indications & technique	3.67	3.73	23.2%	54.5%	Important
Complications of surgery: Managing chronic post-operative swelling	3.67	3.67	20.7%	56.8%	Important
Complications of surgery: Managing neurovascular injuries	3.66	3.53	21.6%	56.8%	Important
Tendon disorders: Arthroscopic flexor hallucis longus (FHL) transfer for chronic Achilles rupture	3.65	3.80	22.8%	53.5%	Important
Degenerative disorders / osteoarthritis: Arthroscopic fusion of talocrural joint (anterior & posterior approach)	3.65	4.14	20.0%	55.5%	Important
Complications of surgery: Single-stage revision	3.65	3.67	23.0%	56.6%	Important
Complications of surgery: Arthrodesis	3.65	3.73	20.2%	57.9%	Very Important
Loose bodies and fractures: Arthroscopic management of ankle fractures (including syndesmotic repair)	3.63	3.93	25.9%	56.3%	Important
Osteochondritis dissecans (OCD): Arthroscopy of talonavicular joint, abrasion & microfracture	3.58	3.27	25.2%	51.3%	Important
Degenerative disorders / osteoarthritis: Arthroscopy +/− osteotomy of talocrural joint (supramalleolar & calcaneal)	3.56	3.73	17.1%	53.2%	Important
Nerve entrapment and injury: Neurolysis	3.55	3.60	17.4%	52.2%	Important
Complications of surgery: Vacuum dressing	3.55	3.57	27.3%	50.9%	Important
Tendon disorders: Tendoscopy of the Achilles tendon	3.49	3.80	19.0%	45.5%	Important
Tendon disorders: Peroneal / tibialis posterior tendoscopy	3.49	3.67	15.4%	45.3%	Important
Loose bodies and fractures: Arthroscopic removal of talonavicular loose bodies	3.47	3.27	18.8%	48.2%	Important
Tendon disorders: Flexor halluces Longus (FHL) release / muscle belly resection for low-lying muscle belly (LLMB) of peroneus brevis	3.39	3.93	14.9%	43.8%	Important
Osteochondritis dissecans (OCD): Arthroscopy of 1st metatarsophalangeal joint, abrasion & microfracture	3.35	3.27	20.4%	43.4%	Important
Degenerative disorders / osteoarthritis: Arthroscopic talonavicular joint fusion	3.33	2.80	10.8%	45.0%	Important
Nerve entrapment and injury: Repair of nerve injury	3.26	2.87	17.5%	41.2%	Important
Loose bodies and fractures: Arthroscopic removal of 1st metatarsophalangeal loose bodies	3.24	3.27	15.9%	40.7%	Important
Degenerative disorders / osteoarthritis: Arthroscopic 1st metatarsophalangeal joint fusion	3.13	2.67	11.9%	38.5%	Important

Survey ratings of all 285 competencies, ordered by overall Mean rating for each specialist area, together with the subgroup Mean rating of specialists in that area, the percentage of 5 and 5 + 4 ratings, and the resulting Level of importance of each competency

### Free text analysis

There were relatively-few free-text responses, most of which were brief and statements rather than suggestions. These included some positive free-text comments about the draft competencies, such as, “Great idea – happy to support”, “It is very detailed very good work”, and “My opinion is that you have covered all important issues”. Several wrote that they had no additional comments, and two indicated that the questionnaire seemed too long. Each expert group reviewed all the free-text comments, paying particular attention to those relating directly to their specialist areas, and agreed there were no new competencies or changes to existing competencies which needed to be incorporated. They felt that some suggestions were already represented in the framework (e.g. “Rupture distal biceps tendon”), others were either too new and experimental (e.g. “Regenerative medicine options”) or no-longer popular (e.g. “Resurfacing arthroplasty of the shoulder”), and some were out-with the scope of the ESSKA competency framework (e.g. “Ethics” and “FIFA protocols”).

## Discussion

### Main findings of this study

The primary finding of this study was the successful development of an evidence-based framework of core treatment competencies for a specialist working in the areas of Knee, Shoulder, Elbow & Forearm, Foot & Ankle and Hip surgery, as well as Sports Medicine. Achieving the aim of this study also supports and allowed the membership to reflect-upon and offer feedback on, ESSKA’s educational strategy and proposed future development more generally. The research involved senior experts in the ESSKA Board, Sections and Committees as well as the wider membership of the society representing 23 countries and a broad range of ages and levels of experience. It also established the level of importance attributed to each of the different competencies by ESSKA members and senior area specialists. The combination of sequential literature review and expert group opinion from area specialists, stakeholder survey and then review and review and incorporation of the results by the expert groups again is a powerful one. To our knowledge, this is the first scientific approach to define and gain consensus on Core Competencies in these specialist areas and, like the ISTH methodology which helped inform but ultimately was different to the current study [11], it is hoped that others international specialist societies may find this approach helpful.

There is significant variability in the way in which specialist in orthopaedics and sports medicine are trained around Europe and the World [16]). Whilst there are some influential and very useful postgraduate training curricula in areas very relevant to ESSKA specialists [3, 13–15], these have been designed as training curricula. They include both additional information which does not need to be defined for ESSKA specialists (such as the basics of patient care and surgical management, or specialist areas out-with the focus of ESSKA), and lack detail in many of the competencies which ESSKA specialists will typically only achieve after completion of specialist training.

### Limitations of this study

Because of the very uneven demographics of respondents between for example Knee and Elbow & Forearm surgeons (which reflects the ESSKA membership), the relatively low response-rate particularly in specialist areas which are under-represented in the ESSKA membership, and the varied interest patterns and areas of expertise leading to most respondents understandably leaving whole subject areas of the questionnaire blank, it was felt that it would not be appropriate to undertake more detailed statistical analysis of correlation coefficients or measures of consensus as has been done elsewhere (e.g. [8, 11]. It was also postulated that the smaller number of area specialists compared to non-specialists rating competencies in some areas might skew the overall Mean and Level to which the competencies were allocated. It was therefore reassuring to find that there were only three competencies where the Mean rating of area specialists was more than one point of difference from the Mean of all responses, and the first of these (3rd in Elbow & Forearm) would clearly have made no difference as a Mean of 5 would have been allocated ‘Essential’ anyway. The second (last in Elbow & Forearm: “Ulnohumeral osteoarthritis: Arthrodesis”) had a Mean of ‘Limited Importance’ from area specialists and more of a candidate for removal from the framework, although it was felt the subgroup of specialist area respondents was really too small on which to base such a decision at this stage. The third (17th in Hip: “Bony deformities: Subspine impingement decompression”), however, was rated significantly higher by the area specialists and could have been moved up two Levels from Important to Essential. It is not clear why there was such a discrepancy, but it is an interesting area for further discussion and research. Also, as highlighted in the free-text comments, the survey was longer than we would have hoped, and it may be that there was an element of ‘survey fatigue’ which led to the first Sports & Exercise area gathering greater responses than the later areas of the survey – at least from respondents who did not indicate that they were primarily specialists in that area.

### Implications for practice and further research

This curriculum project will form the basis for an evidence-based restructuring of ESSKA’s educational activities to support the achievement of these core competencies needed to practice within the various ESSKA specialist areas. This process will involve restructuring and adding surgical skills courses, and mapping relating content in ESSKA’s educational platform: “The ESSKA Academy” [1], to support both theoretical and practical training in the ESSKA Core Competencies. This research has also highlighted a number of areas for future development, not least how and when each of these competencies can best be learned and assessed. The ESSKA core curriculum can also be used by other societies and educational providers to strengthen their educational activities.

## Conclusion

A modular framework of 285 core competencies for ESSKA specialists across all six key specialist areas was developed through a systematic and scholarly approach, involving both expert opinion and engagement of the wider ESSKA membership. The importance of these competencies was reflected in the high ratings attributed to them by society members. This addresses the identified need in terms of defining a core curriculum and ongoing mapping and educational development activities for ESSKA and should also be of interest to the wider orthopaedic and sports medicine communities.

## Data Availability

Anonymised spreadsheets of the data can be made available on request.
